# Cultivating Agents of Change in Medical Students: Addressing the Overdose Epidemic in the United States Through Enhancing Knowledge of Multimodal Pain Medicine and Increasing Accessibility via Open-Access, Web-Based Medical Education and Technology

**DOI:** 10.2196/46784

**Published:** 2023-07-25

**Authors:** Julia H Miao

**Affiliations:** 1 Renaissance School of Medicine at Stony Brook University Stony Brook, NY United States

**Keywords:** medical education, overdose epidemic, opioid epidemic, pain medicine, pain management, opioid use disorder, open-access, telemedicine, teletherapy, technology, public health, opioid, substance use, substance abuse, overdose, SUD, substance use disorder, analgesic, pain, medication management

## Abstract

Medical students of today will soon be physician leaders and teachers of tomorrow about important relevant topics including the overdose epidemic and its devastating impact on our society. In the United States, the overdose crisis, including drug opioid–related overdoses, the increasing prevalence of opioid use disorder along with the increasing number of patients with chronic pain are intensifying and call attention for nationwide action. A strong medical educational foundation of the understanding of the relationship between pain and substance use disorder, their treatment including opioid analgesic therapy, multimodal and interdisciplinary care, and long-term management is needed to help cultivate comprehensive knowledge and training to prepare the next generation’s frontline practitioners to meet these needs. Yet, traditional educational curricula covering these topics are not standardized in medical schools across the nation in the United States. The advent of web-based medical education and the integration of this technology may offer potential solutions to these challenges. Often found equally effective as in-person learning, web-based medical education through open-access modules and other technologies can help increase accessibility, enhance knowledge of multimodal pain management, safe and effective use of opioid analgesics, and other related topics, and provide flexible and powerful teaching initiatives. Our viewpoint is thus that open-access modules and other technology-integrated teaching initiatives can help deliver excellence in pain education, preparing and empowering medical students—our future agents of change—who will be at the forefront of the overdose epidemic.

## Introduction

### Background

With over 3 million people in the United States struggling with opioid use disorder (OUD), the intensifying overdose epidemic calls attention for nationwide action [1]. In 2021, drug overdoses took the lives of over 107,000 people in the United States [2]. Over 80% of those overdose deaths involved opioids, such as fentanyl, prescription opioids, and heroin [3]. An increased number of families across the country have personally witnessed the ravages of the overdose epidemic affecting their loved ones and communities. Recognizing the urgency of this public health crisis and taking action, medical schools and teaching hospitals have started to integrate opioid education into their curriculum to prepare the next generation of health care professionals against this tide.

Medical students, interns, and residents will soon become the front lines of battling this epidemic, treating and managing pain in diverse patient populations. Empowered with the right knowledge, training, and decision-making tools, these providers—teachers of tomorrow and agents of change—can recognize opportunities to make a positive difference in patients’ lives regarding substance use disorder (SUD) and pain management. Just like the incorporation of antibiotic use and microbial resistance into medical curriculum and now the safer practice of antibiotic stewardship in current physician practices, it is important to recognize the need for a strong longitudinal medical foundation for the understanding of both acute and chronic pain and OUD along with their assessment and treatment in order to tackle the seemingly ever-growing epidemic and promote safer opioid practices.

According to the Association of American Medical Colleges (AAMC), a sufficient curriculum on pain medicine and SUD addresses four broad domain areas: (1) the nature of pain; (2) pain assessment; (3) the management of pain, including both treatment and mitigating the risk of overdose; and (4) the relationship between pain and OUD [4]. While medical schools are integrating curriculum on opioid education, a recent study by the AAMC found that only 87% of medical schools in the United States who responded in 2018 covered all 4 topics [4]. In other words, at least 1 out of 10 medical students missed out on learning key competencies of their pain education. Moreover, because of the relatively new implementation of such curricula, the curriculum on opioid use and pain management varies from one school to another and has not been standardized. These challenges thus raise important questions about how we can increase the accessibility of the curriculum for those medical students who may not have had the opportunity to learn certain core competencies or who seek more than what their curriculum includes.

With the COVID-19 pandemic still looming over us, it is also important for us to question what we can do to combat the increasing threat of dual public health crises, where social distancing and continued effective teaching on opioid education are both necessary. During the COVID-19 pandemic, the application of technology and web-based teaching came to the forefront and illuminated the efficacy of web-based medical education. They offered flexibility and feasibility in times of global challenge [5]. In a recent study by Sun et al [6], it was demonstrated that medical students had increased competency and knowledge on opioid education and pain management, equally significant and positive in both web-based and in-person curricula. Other research studies also highlight that there was no significant difference between training modalities (in-person vs web-based) on medical students’ opioid overdose awareness and reversal training (OOART) [7,8]. This demonstrates the tremendous potential that web-based curriculum resources can play a role in enhancing medical students’ opioid and pain knowledge nationwide. With these advantages and equivalent levels of effectiveness as in-person traditional learning, open-access, web-based learning, and medical-educational technology thus may offer solutions to current opioid education challenges. They may not only increase accessibility and enhance the curriculum in areas of a missed opportunity but also provide flexibility and unique web-based learning initiatives that take advantage of effective technology while continuing to deliver excellence in pain education.

This paper, therefore, identifies key web-based medical education resources on pain education in the United States, which not only highlights a collection of currently available and open-access, web-based modules on pain education that can empower medical students but also addresses unique initiatives that take advantage of available technology and effectively improve teaching on topics such as (1) the biopsychosocial model of SUD, (2) breaking down stigma, (3) opioid overdose, and (4) opioid prescribing and pain management. These effective medical education initiatives, integrated with web-based learning and technology, can thus help empower the next generation of medical leaders of the overdose epidemic.

### The Biopsychosocial Model of SUD: Open-Access Modules

In total, 1 out of every 5 American adults experiences chronic pain, affecting over 50 million people [3]. Pain worsens a patient’s quality of life, from impacting sleep and mental health to increasing risk for comorbidities of illnesses and substance use. One of the first competencies of pain education in medical school is to understand the pathophysiology of pain and to understand that SUD is a treatable disorder. Among the many models elucidating pain, the biopsychosocial perspective is the most saliently recognized, illuminating that pain is not simply a physical disorder of peripheral nociception but a dynamic nuanced interaction among biological, psychological, and social factors [9]. Traditionally, medical schools have used in-person lectures to teach about this key area. However, the advent of web-based modular learning ushered in a new way to promote meaningful learning and formative evaluation on opioid education while also ensuring equity of access to content.

Compared to daily web-based lessons, modules provide multiple structured lessons with videos or web-based sessions that can be accessed and completed by a medical student at his or her own learning pace. With clear objectives, modules continue to connect learning goals and can integrate formative testing as a foundation for ongoing evaluation and assessment. Through these videos and web-based modules, medical students can gain a heuristic understanding of the biopsychosocial concept of pain, learning through various organized modules about the interactions of biological, social, and psychological elements unique to the pain of each individual patient.

Open-access, comprehensive web-based modules may offer the solution to fill in gaps of clinical knowledge and understanding. In fact, several open-access e-learning modules from nationally recognized initiatives include but are not limited to the National Neuroscience Curriculum Initiative, Boston University School of Medicine’s Scope of Pain, University of Texas at Austin Dell Medical School Reducing Stigma Education Tool Modules, National Institute on Drug Abuse Modules, and Harvard Medical School’s Opioid Crisis Modules [10-14].

It is also important to note that while open-access modules can help enhance knowledge on the pain curriculum, the sheer abundance of modules that are available and aimed for a wide range of learners at a continued medical education level can make it challenging to choose modules most appropriate for medical student learners. Another challenge is how medical school educators can integrate resources, such as the open-access modules, into an already packed medical curriculum to ensure more standardized teaching across institutions. A cornerstone of medical school curriculum is to foster independent self-directed learning while also avoiding curriculum overload by ensuring appropriate content and sequencing of educational experiences [15]. Therefore, integrating appropriately selected and efficient open-access modules, timed closely with similar clinical or learning experiences, can help best reinforce education and supplementation for medical students.

Among the many current modules available, the following modules have been selected for their appropriate content aimed for an audience of medical students (Table 1); these modules are all open-access and freely available worldwide, allowing more medical students, who may not have a standardized curriculum fulfilling all AAMC competencies at their home institution, to have access to an excellent education on the opioid crisis. While the amount of web-based medical education resources on the overdose epidemic, pain treatment, and management can be overwhelming, these modules provide succinct education with evidence-based medicine on topics from the pathophysiology of pain to its biopsychosocial perspective. The implementation of web-based, open-access modules can also help serve as a continued longitudinal curriculum, either refreshing or advancing medical knowledge and skills in budding medical trainees for lifelong learning.

**Table 1:** Quality and ease of access of selected open-access web-based modules.

Module program	Quality or summary	Ease of access
National Neuroscience Curriculum Initiative	Excellent web-based modules on the neurobiology of acute and chronic pain and SUD^a^ Includes appropriate role-plays and clinical vignettes	Open-access Web-based modules
Boston University School of Medicine’s Scope of Pain	Multidisciplinary resource on the management of acute or chronic pain with opioid analgesics and other treatments More appropriate for higher-level medical students	Open-access Web-based modules Free podcast series Live webinars
University of Texas at Austin Dell Medical School ReSET^b^ Modules	Excellent resource on identifying and addressing OUD^c^-related stigma and biases Appropriate for medical students at all levels	Open-access Web-based modules
Harvard Medical School’s Opioid Crisis Modules	Excellent resource focused on (1) understanding SUD; (2) identification, counseling, and treatment of OUD; and (3) collaborative care approaches for the management of OUD	Open-access Web-based modules
National Institute on Drug Abuse Modules	Excellent resource appropriate for medical students at all levels with multiple modules on (1) introduction to the opioid crisis, (2) treatments for opioid SUD, (3) naloxone access, and (4) biopsychosocial model of SUD	Open-access Web-based modules

^a^SUD: substance use disorder.

^b^ReSET: Reducing Stigma Education Tool.

^c^OUD: opioid use disorder.

### Breaking Down Stigma: Group Teletherapy Visits and Web-Based Objective Structured Clinical Examinations

Patients with OUD and those with SUD often face stigma and negative bias. These stigmatizing perceptions create barriers for patients to seek treatment and access care, further worsening their health outcomes and road to recovery [16]. It is imperative for clinicians to thus reduce stigma and enhance care for patients with OUD. However, one study found that only 20% of general internists surveyed reported feeling prepared to screen individuals with SUD [17]. Even so, 30% of the surveyed internists perceived patients with OUD as different from those with chronic illness [17]. These perceptions may derive from inadequate training and medical education on OUD in health care professionals.

Medical schools recognize the potential negative impact that stigma may have and have stepped in to break down stigma and these perceptions by facilitating medical students to work with patients with OUD directly and to see the effects of opioid SUD first-hand in communities. For example, medical students attend 12-step programs or team therapy sessions in-person to listen to the stories of individuals recovering from OUD, who gather together in groups to share their feelings, thoughts, and progress [18]. They see the ravages of opioid SUD first-hand and apply the lessons they learn in the classroom to these intimate group sessions. These opportunities humanize SUD and the people who experience it. They also give students a greater sense of responsibility as a physician.

However, with the COVID-19 pandemic and the necessity for safe social distancing, there was a need to create web-based approaches to continue these unique initiatives. One example is web-based group therapy sessions, also known as group teletherapy. Just like e-learning was found to be equally effective as in-person learning for pain education, group teletherapy was also found to be equally effective as in-person therapy for patients with OUD [19,20]. Likewise, medical students can continue to listen to the stories of patients with OUD through visiting group teletherapy sessions. Safe web-based platforms such as Teams (Microsoft Corp) have ensured security and confidentiality. These initiatives continued to help medical students to break down stigma and possible inherent biases and foster compassion for their patients and community members facing OUD or SUD.

Evidence-based educational modules also used web-based platforms to address OUD-related stigma. One of the most prominent examples is the Dell Medical School’s Reducing Stigma Education Tools, which are excellent open-access resources that teach about reducing stigma in people with OUD, covering clinical application, patient-centered management for recovery, and motivational interviewing [12]. Other applications of medical education technology include the increasing use of web-based objective structured clinical examinations (OSCEs). One medical school’s longitudinal pain and SUD curriculum incorporated a web-based OSCE over Zoom that included 3 patient vignettes involving patients requesting for an early refill of opioid medication [21]. Through these “teleOSCEs,” students are taught to recognize the impact of their personal biases on medical decision-making with patients with pain or those with SUD [21]. This is just one example highlighting how web-based OSCEs were thus effective in helping promote reflection for change.

Both web-based opportunities, such as teleOSCEs and group teletherapy on breaking down OUD-related stigma, however, are not without their own limitations. Incorporating and assessing physical examination skills in web-based settings may be challenging, but OSCEs with the objective to evaluate and reflect on one’s own personal OUD-related biases can be carefully formatted to integrate more practice and assessment on interpersonal communication and management rather than physical examination skills. Additionally, a disadvantage of group teletherapy is the lack of physical presence—these group therapy sessions often occur where patients gather closely together in a circle, offering a unique connection for one another, which may be otherwise reduced digitally. Nevertheless, web-based formats such as these offer beneficial alternatives for enhancing reflection, overcoming public health barriers, and opening up opportunities for future studies to adopt more feasible hybrid formats [22].

### Opioids and Opioid Overdose: OOART and Simulations

Vital aspects of a pain curriculum include learning opportunities on opioid overdose and its treatment. Many medical schools have incorporated OOART early on in their curriculums, introduced often to first-year medical students to address misconceptions and gaps in opioid overdose knowledge [23]. However, OOART is often taught in a large classroom-based, one-time session, calling attention to the need for longitudinal posttraining retention and assessment.

Two independent analyses found that web-based OOART was found to be equally effective as in-person sessions [7,8] and with the additional benefit of facilitating longitudinal posttraining assessment. Various teaching institutions have found the application of simulation with web-based mannequins and technology to be effective teaching tools for students to apply their post-OOART knowledge as well. Simulations tested team-based learning in an emergency scenario, assessing for communication, teamwork, critical thinking, and clinical decision-making skills. Common simulation scenarios involve an unconscious patient with OUD, the unresponsive mannequin. These scenarios challenge medical students to apply their clinical knowledge and skills to real-time situations. More importantly, postsimulation reflections and teaching improved students’ understanding of the appropriate clinical management during patients’ treatment period that can potentially lower their risk for future abuse, misuse, or diversion of opioids.

While technology and web-based medical-educational resources have become increasingly prevalent in medical education, there are also potential limitations to their integration when it comes to teaching pain curricula on the overdose epidemic. One significant limitation is the difficulty in simulating the tactile experience of diagnosing and treating pain in a web-based setting. This can hinder the development of essential skills such as physical examination and palpation techniques. Especially in OOART, hands-on experience, such as performing the physical intervention of rescuing a patient with OUD, is important. However, in-person simulations with technology-integrated, hands-on mannequins may help to bridge these gaps. As such, it is essential to balance the benefits of technology with in-person, hands-on training to ensure that medical students receive a comprehensive education on pain medicine and the overdose epidemic.

### Opioid Prescribing and Pain Management: Teaching in Telemedicine

Opioid prescribing and pain management continue to remain essential in the pain curriculum with a high level of relevance in digitally all fields of medicine, from anesthesiology and surgery to general medicine and interventional radiology. Opioid analgesic therapy, however, is only one part of a comprehensive multimodal pain treatment plan, which must also be covered in a comprehensive curriculum as well. What makes pharmacologic treatment particularly challenging is that while it can help reduce pain and adverse events, sometimes, the treatment modalities themselves are not without their own morbidities, side effects, and risks that can even worsen patient outcomes, especially for more vulnerable populations such as children, adolescents, and the elderly.

Clinicians have the duty to prevent unnecessary discomfort, optimizing effective pain management. Therefore, pain curricula in medical schools and teaching hospitals have been adapted to teach additional nonpharmacological treatments, including cognitive behavioral therapy and physical therapy (PT) for patients with acute or chronic pain or those with OUD [24]. Most recently, pedagogical approaches have integrated new technology, including telemedicine, to effectively reinforce concepts of the pharmacological and nonpharmacological aspects of pain management. In telemedicine, medical students have the opportunity to make a direct impact on real patients with acute and chronic pain and OUD by contributing to clinical decision-making with their preceptors.

However, similar to the challenges of a web-based OOART, there remain limitations to teaching in telemedicine, including the difficulty of providing essential tactile and physical examination experiences for students interacting with various pain management techniques. For example, alternate nonpharmacological treatments, such as PT and pain intervention, are challenging to experience directly. Additionally, in-person experiences with meeting and treating patients with chronic pain or SUD are invaluable, involving emotional and social complexities and interpersonal communication that may be otherwise missed digitally.

Nevertheless, through active participation in clinical management over telehealth, students see the combination of various therapies in action, including opioid and neuropathic pain medications, cognitive behavioral therapy, and PT, among others. With additional benefits of social distancing for public health and safety, enhanced accessibility with no geographic or cost limitations, as well as synchronous telehealth accommodating multiple students at the same time, these technologically integrated opportunities can further enhance the knowledge, accessibility, and skills of medical students and future health care providers to treat patients with OUD across the nation.

## Conclusions

Building a strong medical educational foundation on pain knowledge and multimodal management is essential in training the next generation of physicians at the front lines of the overdose epidemic. However, the pain curriculum in training programs, particularly in the United States, has frequently been challenged with accessibility and standardization issues. Unique teaching initiatives integrating effective medical education technologies and web-based resources have been innovated to help solve some of these challenges, including open-access modules, telemedicine, web-based OSCEs, simulations, and group teletherapy visits. Open-access modules can be an especially powerful tool that can increase medical students’ accessibility to more learning opportunities and a comprehensive pain curriculum beyond their medical schools from learning about the biopsychosocial model to engaging in OOART training. The flexibility of web-based OSCEs, telemedicine, and group teletherapy visits also is advantageous in breaking down the stigma of OUD and fostering compassion. Web-based OSCEs and simulations have the ability to strengthen communication skills, foster interprofessional teamwork, and enhance critical thinking and clinical decision-making skills by helping to apply medical students’ knowledge to opioid overdose and management. While this paper does not exhaust all existing resources, it highlights key web-based medical-educational resources and the positive impact integrated technology has on the pain curriculum and its increasing significance in the overdose epidemic. Our opinion is thus that open-access modules and other technology-integrated teaching initiatives can help effectively improve and teach key competencies of the pain curriculum and empower medical students—our future agents of change—with the knowledge and confidence needed to address this public health crisis.

## Abbreviations

AAMC: Association of American Medical Colleges
OOART: opioid overdose awareness and reversal training
OSCE: objective structured clinical examination
OUD: opioid use disorder
PT: physical therapy
SUD: substance use disorder
